# Low-field magnetic resonance imaging study on carpal arthritis in systemic sclerosis - low-grade erosive arthritis of carpal bones is an unexpected and frequent disease manifestation

**DOI:** 10.1186/ar4128

**Published:** 2013-01-04

**Authors:** Elif Akbayrak, Robert Dinser, Ulf Müller-Ladner, Ingo H Tarner

**Affiliations:** 1 Department of Internal Medicine and Rheumatology, Justus-Liebig-University Giessen, Department of Rheumatology and Clinical Immunology, Kerckhoff-Klinik, Benekestraße 2-8, D-61231 Bad Nauheim, Germany

## Abstract

**Introduction:**

The aim of the present study was to assess the prevalence and characteristics of subclinical arthritis of carpal and metacarpophalangeal joints in patients with systemic sclerosis (SSc).

**Methods:**

Low-field (0.2 T) magnetic resonance imaging (MRI) was performed in consecutive patients with SSc attending our center between January 2010 and March 2011. Results were assessed in a standardized manner using the Rheumatoid Arthritis Magnetic Resonance Imaging Score (RAMRIS) and standardized assessments of all hand joints. Patients with arthritis due to overlap syndromes were excluded.

**Results:**

Of 38 inpatients and eight outpatients who were screened for inclusion, 30 patients participated in the study and 26 patients could be evaluated. Erosions, bone marrow edema, synovitis, and joint effusions were found in 87%, 37%, 68%, and 58%, respectively, and 24% of patients had additional tenovaginitis. Arthritis affected only a low number of joints per analyzed hand. All bones and joints could be affected, but synovitis and bone marrow edema occurred predominantly in the proximal row of carpal bones, most frequently affecting the lunate bone. The extent of inflammatory changes measured with the RAMRIS correlated significantly with the functional status assessed with the validated German functional score questionnaire Funktionsfragebogen Hannover.

**Conclusion:**

Low-grade arthritic changes on low-field MRI are frequent in patients with pure SSc. The features of arthritis in SSc differ from rheumatoid arthritis. The distribution, the MRI pattern and the predilection for the lunate bone raise the hypothesis that arthritis in SSc may be caused not only by immunological inflammation but also by ischemic mechanisms.

## Introduction

In the clinical evaluation of systemic sclerosis (SSc), attention is predominantly given to changes of the skin, Raynaud's phenomenon and its complications, and internal organ involvement. While arthritis has been observed in SSc [1], it is frequently considered to indicate an overlap between rheumatoid arthritis (RA) and SSc [2,3], whereas joint pain in patients with sole SSc is commonly regarded as non-inflammatory arthralgia caused by skin tightness and flexion contractures [4].

Register studies [5,6] and retrospective clinical studies including a meta-analysis of existing data [7] have recently suggested that inflammatory arthritis may be an underestimated problem in SSc. This suggestion could be of therapeutic importance because disease-modifying antirheumatic drugs available for inflammatory arthritis might also be useful to delay or prevent joint damage and loss of function in SSc patients.

We therefore performed a prospective magnetic resonance imaging (MRI) study of the hands in patients with SSc, excluding patients with clinical or immunological signs suggesting an overlap with other forms of arthritis. We aimed to determine the prevalence of MRI signs of arthritis including synovitis, bone marrow edema, effusions, and erosions as well as to characterize the distribution of joint involvement. MRI was chosen because it is an imaging tool with high sensitivity for the detection of inflammatory joint changes, as has been demonstrated very well in RA [8] and also in SSc [9,10].

## Materials and methods

Consecutive patients with SSc treated as inpatients or outpatients in our department between January 2010 and March 2011 were considered for this study. Patients had to fulfill the LeRoy criteria for limited cutaneous SSc or diffuse cutaneous SSc [11]. We excluded patients with the clinical picture of an overlap syndrome (such as Sharp's syndrome) or a clinical association with other rheumatic diseases such as ankylosing spondylitis or RA. We also excluded patients in whom antibodies against U1-ribonucleoproteins or anti-citrullinated peptide antibodies (ACPA) had been detected either within the previous year or when screened for enrolment. Outpatients had to live within a radius of 50 km to be able to return for the study assessments. To address the problem of subclinical arthritis, the presence or absence of joint pain or swelling was not considered for inclusion.

After obtaining written informed consent, the following assessments were performed: tender and swollen hand and finger joint counts; full skin status using the modified Rodnan skin score; and functional assessment using the German-language questionnaire Funktionsfragebogen Hannover (FFbH) with values ranging from 0 to 100, the latter reflecting completely normal function [12]. Use of the FFbH is standard at our center and FFbH values can be converted into health assessment questionnaire (HAQ) values using the formula:

$$\text{HAQ }=\;3.16\;-\left(0.028\times \text{FFbH}\right).$$

The calculated HAQ is therefore also presented [13]. The erythrocyte sedimentation rate and, if not documented previously, ACPA were measured. Morbidity and SSc-related treatments were also recorded.

Patients underwent low-field MRI (0.2 T, Esaote C-scan; Esaote, Cologne, Germany) of the carpus and the metacarpophalangeal joints in either the more painful hand or, if not applicable, the dominant hand [14,15]. A three-dimensional, gradient-echo T1-weighted sequence, a fat-saturated short-tau inversion recovery T2-weighted sequence, and a second three-dimensional, gradient-echo T1-weighted sequence were acquired after application of a gadolinium-based contrast agent (Gadodiamide, Omniscan™; GE Healthcare, Munich, Germany). In consenting patients, the other hand was measured after a time interval of at least 1 day to allow for complete washout of the contrast agent.

Images were assessed systematically using two approaches. In the first approach the Rheumatoid Arthritis Magnetic Resonance Imaging Score (RAMRIS) [16,17] developed for RA and high-field MRI was applied by two independent investigators, and scores were calculated using the average score of both investigators for each item. Owing to the technical lack of fat suppression for T1-weighted images in low-field MRI, synovitis was assessed by comparing signal intensities of the synovium before and after the application of Gadodiamide side by side. Thickening of the synovium with enhancement after application of the contrast agent was judged as synovitis, which was assessed only if contrast agent could be applied. Erosions were defined to be present if the contour of the cortical bone was interrupted in at least two anatomical planes and if enhancement of the defect with Gadodiamide was detected. In series without application of contrast agent, the first criterion was deemed sufficient. The presence of bone marrow edema was scored as definite if a hyperintense signal could be detected in the short-tau inversion recovery sequence image and a hypointense signal could be detected in the T1-weighted image.

As the RAMRIS does not judge effusions and preselects joints involved in RA, a second descriptive systematic analysis for the presence of synovitis, bone marrow edema, erosions, joint effusion, and tenovaginitis was performed for the following areas: radiocarpal, ulnocarpal, radioulnar, intercarpal and first carpometacarpal joints as well as metacarpophalangeal joints 1 to 5. For bone marrow edema and erosions, bones of the carpus and metacarpus were analyzed separately (distal ulna, distal radius, scaphoid, lunate, triquetral, pisiform, trapezium, trapezoid, capitate, hamate, bases of metacarpal bones 1 to 5, heads of metacarpal bones 1 to 5, and bases of proximal phalanges 1 to 5). Flexor and extensor tendons were analyzed for tenovaginitis.

Each MRI feature was classified as absent or questionable, clearly present, or severe. If the increase in signal intensity after contrast injection was obvious even in the absence of a comparison with the baseline image, synovitis was classified as severe. Severe erosions were defined as defects occupying more than 50% of the bony surface. Effusions were analyzed in the short-tau inversion recovery sequence, with a convex contour of the fluid signal within the joint capsule considered definite, and a distention of the overlying skin contour considered severe. Bone marrow edema of more than 50% of the existing bone area was considered severe. Examples for typical findings for each assessment are shown in Figure S1 in Additional file 1.

For statistical analysis, the Wilcoxon rank test or Fisher's exact test were used where appropriate. Correlations were analyzed using Kendall's tau rank test [18], which is similar to Spearman's Rho rank correlation test but does not imply a linear correlation between values.

The study was approved by the ethics committee of the University of Giessen.

## Results

A total of 52 inpatients were screened for enrolment. Four patients were excluded due to overlap with other rheumatic diseases, and three patients were excluded because their clinical condition did not permit MRI measurements. Seven patients were unable to participate due to organizational difficulties. Of the 38 inpatients enrolled in the study, 27 agreed to undergo MRI measurements in one or both hands. Eight outpatients were also found to be eligible, three of whom agreed to participate.

Three patients had to be excluded from the analysis after acquisition of MRI measurements, because positive results for ACPA became available in two of them and the diagnosis had to be revised to Sharp's syndrome in the third patient. The signal quality of the MRI measurements was inadequate for interpretation in one patient.

A total of 26 patients could therefore be analyzed, of whom 12 agreed to measurements in both hands and the other 14 only to measurements in one hand. A total of 38 hands could thus be examined. Owing to difficulties in obtaining venous access, contrast agent could not be applied in seven of the 38 MRI examinations. None of the patients fulfilled either the current American college of Rheumatology/European League Against Rheumatism or the former American College of Rheumatology classification criteria for RA [19,20].

The characteristics of our group of patients and a summary of the results, including the RAMRIS and the functional FFbH and HAQ scores, are shown in Table 1. The detailed RAMRI scores for each patient are presented in Table S1 in Additional file 2.

**Table 1:** Patient characteristics

Parameter	All SSc (*n *= 26)	Diffuse cutaneous SSc (*n *= 6)	Limited cutaneous SSc (*n *= 20)
Age (years)	56 ± 13 (32 to 75)	50 ± 9 (40 to 66)	46 ± 14 (32 to 75)
Sex (% female)	77	67	80
Disease duration (years)	7.9 ± 5.3 (1 to 19)	7.7 ± 6.0 (3 to 19)	8.0 ± 5.2 (1 to 18)
Modified Rodnan skin score	8.3 ± 6.1 (2 to 19)	16.3 ± 3.3 (12 to 19)	5.8 ± 4.3^† ^(2 to 18)
Organ and tissue involvement			
Pulmonary fibrosis	11 (42)	6 (100)	5 (25)**
Pulmonary hypertension	5 (19)	3 (50)	2 (10)
Cardiac involvement	6 (23)	1 (17)	5 (25)
Gastrointestinal involvement	2 (8)	1 (17)	1 (5)
Active digital ulcer	7 (27)	0 (0)	7 (30)
History of digital ulcer	16 (62)	4 (83)	12 (55)
Arteriosclerotic disease	3 (12)	0 (0)	3 (15)
ESR (mm/hour)	19 ± 14	28 ± 20	16 ± 11
Antinuclear antibody positivity	23 (89)	5 (83)	18 (90)
Anti-centromere antibody	11 (42)	0 (0)	11 (55)*
Anti-Scl70 antibody	9 (35)	2 (33)	7 (35)
Medication			
Immunomodulatory drug^a^	10 (38)	2 (33)	8 (40)
Steroids	6 (23)	1 (17)	5 (25)
Iloprost	12 (46)	3 (50)	9 (45)
Calcium-channel blockers	9 (35)	2 (33)	7 (35)
Betablockers	1 (4)	0 (0)	1 (5)
Bosentan	7 (27)	3 (50)	4 (20)
Sildenafil	3 (12)	0 (0)	3 (15)
Low-dose aspirin	5 (19)	0 (0)	5 (25)
RAMRIS	6.5 ± 4.7 (0 to 14)	5.4 ± 4.1 (1 to 12)	7.0 ± 5.0 (0 to 14)
Radiographs of the hands	21 (81)	4 (67)	17 (85)
Signs of arthritis	2 (10)	0 (0)	2 (12)
Acroosteolysis	9 (43)	2 (50)	7 (41)
Soft-tissue calcifications	11 (52)	1 (25)	10 (59)
FFbH (% functional capacity)	68.7 ± 22.8 (33 to 100)	58.2 ± 25.2 (39 to 100)	71.9 ± 21.7 (33 to 100)
Health Assessment Questionnaire^b^	1.24 ± 0.64 (0.36 to 2.25)	1.54 ± 0.7 (0.36 to 2.08)	1.15 ± 0.61 (0.36 to 2.25)

Data presented as mean ± standard deviation (range) or *n *(%). Only the Rheumatoid Arthritis Magnetic Resonance Imaging Score (RAMRIS) for the clinically dominantly affected hand was analyzed. Percentages were rounded. ESR, erythrocyte sedimentation rate (Westergren's method); FFbH, Funktionsfragebogen Hannover (German-language standardized assessment questionnaire on physical function); SSc, systemic sclerosis. Significant differences: *P < 0.05 and **P < 0.01 between the groups with limited and diffuse skin disease as assessed by Fisher's exact test; ^†^*P *< 0.01 as assessed by the Mann-Whitney U test. All other comparisons were nonsignificant. ^a^Including methotrexate, leflunomide, mycophenolate, etanercept, and cyclophosphamide. ^b^Values calculated from the FFbH using the formula: HAQ = 3.16 - (0.028 × FFbH)).

At the time of MRI examination, 10 patients received immunomodulatory drug treatments: five patients were being treated with methotrexate, one each with leflunomide, mycophenolate, and etanercept, respectively, and two patients were being treated with cyclophosphamide. All patients on prednisolone took ≤ 5 mg/day. Iloprost (Ilomedin™; Bayer Vital GmbH, Leverkusen, Germany) was applied to 46% of patients at the time of MRI examination because of a clinical worsening of their Raynaud's syndrome. Nineteen patients were on long-term vasodilating drugs (calcium channel blockers, bosentan and sildenafil), and three patients had concomitant arteriosclerotic disease (two coronary heart disease, one peripheral arterial disease).

When analyzing all measured hands, every MRI feature of arthritis was observed in a high proportion of patients (Table 2) even though 64% of all hands analyzed did not exhibit any clinical pain or swelling in the joints assessed by MRI. Only 21% of all hands did not show any signs of current arthritis on MRI (synovitis, effusion, bone marrow edema).

**Table 2:** Proportion of hands showing different features of arthritis on magnetic resonance imaging

	Erosion	Bone marrow edema	Synovitis	Joint effusion	Tenovaginitis
Signs of arthritis					
Not present/indeterminate	13	63	32	42	76
Present	87	37	68	58	24
Severe	3	16	13	0	0
Affected joints in affected hands	2.2 ± 1.6 (1 to 6)	1.6 ± 1 (1 to 4)	4.1 ± 2.6 (1 to 9)	4.2 ± 2.6 (1 to 10)	N/A

Data presented as percentage or mean number ± standard deviation (range). For definition of definite or severe please refer to Materials and methods. N/A, not applicable.

While effusions and erosions were frequent, they were rarely severe (Table 3; for reference, see also Figure S1 in Additional file 1). Strong synovial enhancement was present in 13% of analyzed patients. Severe bone marrow edema occurred in 15% of patients, suggesting a strong association of this feature of arthritis with SSc. In the majority of patients, arthritis affected only a few joints within one hand.

**Table 3:** Distribution of magnetic resonance imaging findings in individual joints and bones of the hand

	Synovitis		Joint effusion	
	
	Definitive	Severe	Definitive	Severe
Joint				
Radiocarpal	19	3	8	-
Radioulnar	23	-	-	-
Ulnocarpal	48	6	-	-
Intercarpal	52	3	47	-
Carpometacarpal joint 1	6	-	3	-
Metacarpophalangeal joint 1	13	-	11	-
Metacarpophalangeal joint 2	6	-	3	-
Metacarpophalangeal joint 3	23	-	-	-
Metacarpophalangeal joint 4	16	-	5	-
Metacarpophalangeal joint 5	16	-	5	-
	
	**Bone marrow edema**		**Erosion**	
	
	**Definitive**	**Severe**	**Definitive**	**Severe**
	
Bone				
Ulna/radius	-	-	3	-
Scaphoid	3	-	13	-
Lunate	18	11	45	3
Triquetral	8	-	50	-
Pisiform	-	-	-	-
Trapezium	5	3	13	-
Trapezoid	-	-	5	-
Capitate	3	-	24	-
Hamate	-	-	3	-
Metacarpal base 1 to 5	2	1	2	-
Metacarpal head 1 to 5	1	-	8	-
Phalangeal base 1 to 5	-	-	2	-

For each magnetic resonance imaging feature, frequencies of definite and severe manifestation are indicated as a percentage. -, not present. Joint effusion, bone marrow edema and erosions could be determined in 38 hands from our 26 patients. Synovitis could only be assessed in 31 hands, due to lack of venous access for the application of contrast agent.

When analyzing the localization of affected joints and bones, synovitis was found more frequently in the intercarpal joints than in the metacarpophalangeal joints (Table 3), with severe synovitis occurring in up to 6% of carpal joint areas. While bone marrow edema could affect most bones of the carpus and metacarpus, it most frequently and most severely affected the lunate, with a definite edema occurring in 18% of lunate bones and severe edema in 11%. The scaphoid and triquetrum were also prone to bone marrow edema. The predilection of the proximal row of carpal bones for bone marrow edema was also reflected in the frequency of erosions in these bones (Table 3). No synovitis could be found in 27% of joint areas with bone marrow edema. None of the patients has had previous severe trauma or fractures of the distal forearm, carpal bones, metacarpal bones, or fingers, and only one patient had a history of occupational exposure to vibratory tools or machinery (a construction worker who frequently drilled concrete). In the latter patient, however, only the lunate and triquetrum of the left hand each showed a small erosion without bone marrow edema whereas the other carpal bones of both hands showed no abnormality.

**Figure 1:**
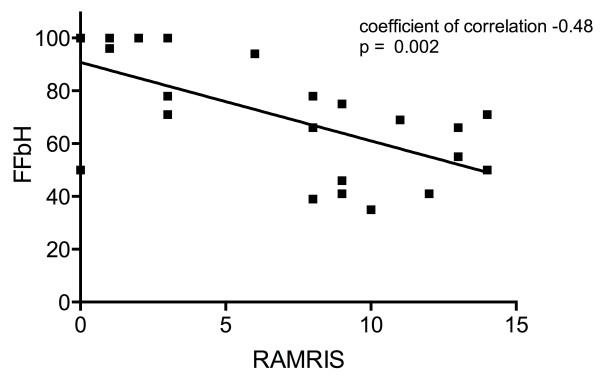
**Correlation of the arthritis score with global function**. Correlation of the Rheumatoid Arthritis Magnetic Resonance Imaging Score (RAMRIS) with global function assessed by the German Funktionsfragebogen Hannover (FFbH) questionnaire.

The RAMRIS validated for RA correlated moderately with the overall functional joint status assessed by the FFbH (correlation coefficient = -0.48, *P *= 0.002; Figure 1) as well as the calculated HAQ scores (correlation coefficient = 0.48, *P *= 0.002). A summary score calculated from the secondary systematic assessment correlated strongly with the RAMRIS (correlation coefficient = 0.80, *P *< 0.0001). Clinical tender or swollen joint count results did not match with MRI findings. The arthritis score was more severe in patients with more widespread disease involvement, reflected by the number of affected organ systems (correlation coefficient = 0.43, *P *< 0.01), but there was no association with the degree of skin involvement as measured by the modified Rodnan skin score, systemic inflammation as assessed by erythrocyte sedimentation rate, disease duration, or age. The RAMRIS did not differ between patients with short disease duration ≤ 3 years and patients with longer-standing disease (mean ± standard deviation RAMRIS, 6.3 ± 4.3 vs. 6.6 ± 5.0, *P *= 0.918). There was also no correlation between immunosuppressive drug treatment, iloprost treatment, long-term vasodilating drug treatment, β-blocker use, low-dose aspirin use, or concomitant arteriosclerotic disease and the presence of MRI features of arthritis. Neither was affection of the lunate bone correlated with arteriosclerosis or the use of vasodilating drugs, β-blockers or low-dose aspirin.

Radiographs of the hands were obtained as part of the diagnostic work-up in 21 of the 26 patients (81%; Table 1). Two patients had radiographic changes of the lunate. One of these patients had a small erosion that corresponded to the erosion seen on low-field MRI. The other patient exhibited small cystic changes of both lunate bones but no definitive erosions on X-ray, whereas low-field MRI clearly showed bilateral lunate erosions. In all other patients with lunate erosions on MRI, no abnormalities were detected by X-ray scan. Of note, none of the patients with bone marrow edema of the lunate bone had radiographic abnormalities. Acroosteolysis and extraarticular soft-tissue calcifications were observed in 43% and 52% of the radiographs, respectively (Table 1). The presence and severity of MRI features of arthritis, however, were not correlated with the finding of either acroosteolysis or calcifications.

## Discussion

This prospective systematic low-field MRI study shows that subclinical arthritis of the carpus and metacarpus is a frequent manifestation in patients with SSc, which is underestimated by clinical examination as well as plain radiographs. The relevance of this observation is underlined by the correlation of a quantitative assessment of arthritic changes, the RAMRIS, with validated functional assessments, the FFbH and HAQ. The arthritis score is also associated with severity of disease estimated by the number of affected organ systems.

The presence of arthritis in SSc has been suggested in large register studies [5,6]. A retrospective cohort analysis with supplementary meta-analysis from our group also supports a high prevalence of clinical and erosive arthritis as a genuine feature of SSc [7]. Register cohorts have the drawback that data are frequently collected by nonrheumatologists, resulting in a high heterogeneity in classification of joint findings [5]. Furthermore, a definite differentiation between overlap syndromes and pure SSc is difficult within register studies, retrospective approaches or meta-analyses [7].

MRI is considered a very sensitive method for the detection of arthritis. High-field MRI has thus far been used to examine hand joints in SSc patients in two smaller studies. One retrospective study on 17 patients with joint pain and SSc observed inflammatory changes in 59% of patients [9]. In this study, bone marrow edema was even more prominent (53%) than in our cohort (37%), whereas the proportion of patients with erosions was much lower (41%) compared with our study (87%). Unfortunately, no details are provided on the localization of bone marrow edema in the different patients [9]. Another prospective study analyzed 17 patients with arthralgias and SSc by ultrasound, eight of whom also underwent MRI [10]. Joint synovitis was found by ultrasound in one of 17 patients initially, in three of 13 patients after 6 months, and in eight of eight patients analyzed by MRI. Of these eight patients, five also exhibited bone marrow edema and six patients had erosions [10]. Ultrasound thus appears to underestimate arthritis manifestations in SSc even in patients with clinical arthralgias.

The high sensitivity of the MRI technique, the relatively large number of patients for a single center and the inclusion of patients independent of clinical arthralgia or joint swelling as well as the stringent exclusion of known arthritis-associated diseases are specific strengths of our study for the determination of a point prevalence of arthritis in SSc in comparison with the other studies. A drawback of our study is the absence of a healthy control group, since erosions can also be observed in healthy subjects [21]. Even though we used a very stringent definition of erosion, the prevalence of erosions may thus be overestimated. On the contrary, the low-field technique underestimates bone marrow edema compared with high-field MRI [14], which may explain the difference in comparison with the results of Low and colleagues [9]. The failure to inject intravenous contrast agent in 18.5% of the 38 MRI examinations leads to underestimation of synovitis in the RAMRIS. The multitude of arthritis-associated MRI findings and the association of quantitative arthritis assessments with the functional score are arguments for the overall validity of our findings. Another drawback is that 35% of eligible patients declined participation, even though the positioning in the low-field MRI is more tolerable than in high-field MRI. The overall disease severity of SSc in these patients was comparable, but this dysbalance may bias our findings.

The prevalence and severity of erosions, bone marrow edema, and synovitis in our cohort with longstanding SSc are comparable with studies on patients with early RA [22]. In longstanding RA, the severity of inflammatory and destructive changes usually increases and affects more joints, thus leading to more severe MRI findings than those observed in our study [8]. Owing to the fact that the patients in our study did not fulfill any classification criteria of RA and that the detected arthritic changes were relatively mild despite long-standing disease without disease-modifying anti-rheumatic drug treatment in the majority of cases, we conclude that arthritis in SSc does not reflect an overlap syndrome with RA [2,3], but represents a genuine disease manifestation.

The high prevalence of bone marrow edema in our cohort is of specific interest since bone marrow edema in RA usually heralds erosions [15,22]. The same indication appears to be true for arthritis in SSc since the finding of bone marrow edema was frequently associated with erosions in our study. The predilection of bone marrow edema and erosions for the lunate and other bones of the proximal row of carpals is noteworthy and reminiscent of early osteonecrosis.

This finding raises the hypothesis that not only an autoimmune process but also ischemia on the basis of the characteristic microangiopathy of SSc and the frequent stenosis of arterial vessels of the wrist in patients with SSc [23,24] may play a role in SSc arthritis and its predilection for the lunate bone. Along this line, four cases have been published [25,26] that illustrate an association between osteonecrosis of the lunate bone and SSc with severe Raynaud's phenomenon, the clinical hallmark of ischemia in SSc. In addition, the severity of Raynaud's phenomenon has been associated previously with the development of erosive arthritis in SSc [27]. The prominent affection of the lunate bone and the high severity of Raynaud's phenomenon in the majority of our patients - as indicated by the high proportion of patients complaining of clinical deterioration (62%), the need for intravenous iloprost despite long-term use of oral vasodilators, and the high proportion of current or previous digital ulcers - thus fit very well with the hypothesis that SSc arthritis is triggered by reactions to ischemia in the context of severe Raynaud's phenomenon [28] in addition to immunological mechanisms.

## Conclusion

In summary, arthritis characterized by mild synovitis, bone marrow edema with a predilection for the lunate bone, mild effusions, and low-grade erosions is a clinically underestimated but frequent, genuine feature of SSc. The pattern of arthritis does not resemble RA. We hypothesize that an ischemic component reflected by severe Raynaud's phenomenon may be a key trigger for this type of joint manifestation. Further studies on a larger number of patients are needed to further verify this hypothesis, which would then open up new avenues for the treatment of arthritis in SSc.

## Abbreviations

ACPA: anti-citrullinated peptide antibodies; FFbH: Funktionsfragebogen Hannover; HAQ: Health Assessment Questionnaire; MRI: magnetic resonance imaging; RA: rheumatoid arthritis; RAMRIS: Rheumatoid Arthritis Magnetic Resonance Imaging Score; SSc: systemic sclerosis.

## Competing interests

The authors declare that they have no competing interests.

## Authors' contributions

EA, RD, and IHT designed the study. EA and IHT performed the examination of patients and the low-field MRI measurements. EA, RD, and IHT analyzed the MRI images, RD and IHT performed the RAMRIS. EA, RD, UM-L, and IHT discussed and interpreted all data. EA drafted the manuscript, and RD, UM-L, and IHT critically reviewed and revised the manuscript. All authors read and approved the submitted manuscript. EA, UM-L, and IHT read and approved the final manuscript after RD deceased.

## Supplementary Material

Additional file 1 **Figure S1 showing typical examples of arthritis features on low-field MRI**. This figure provides typical examples of synovitis, bone marrow edema, erosions, joint effusion, and tenovaginitis on low-field MRI. (A) Synovitis: T1-weighted gradient-echo sequence before (1, 3) and after (2, 4) application of Gadodiamide shows definite (*) synovitis of the intercarpal and radioulnar joints (1, 2) and the third metacarpal joint (3, 4) and severe (arrows) synovitis with synovial thickening and contrast enhancement of the radiocarpal and ulnocarpal joints (1, 2). (B) Bone marrow edema (arrows) affecting < 50% of the bone marrow area (1, 2) and severe edema affecting > 50% of the bone marrow area (3, 4) appear as hypointense areas in T1-weighted images (1, 3) and hyperintense areas in T2-weighted short-tau inversion recovery images (2, 4). (C) Erosion: T1-weighted gradient echo sequence before (1) and after (2) application of Gadodiamide show erosions (arrows) of different sizes. A severe erosion is shown in coronary (3) and transverse (4) sections. (D) Effusion: T2-weighted short-tau inversion recovery image visualizes hyperintense fluid signals (arrows) of normal joint fluid (1), definite effusion (2, 3), and severe effusion (4, 5) as well tenovaginitis of the flexor tendons grade 2 (6). Click here for file

Additional file 2 **Table S1 presenting a summary of the RAMRIS**. This table provides an overview of the RAMRIS per patient. N/A, not applicable due to lack of venous access for the injection of contrast agent. Click here for file
