# Supplementary figures and images for: Low-field magnetic resonance imaging study on carpal arthritis in systemic sclerosis - low-grade erosive arthritis of carpal bones is an unexpected and frequent disease manifestation
Source: Arthritis Res Ther. 2013 Jan 4;15(1):R2. doi: 10.1186/ar4128 (PMC3672762; doi:10.1186/ar4128)

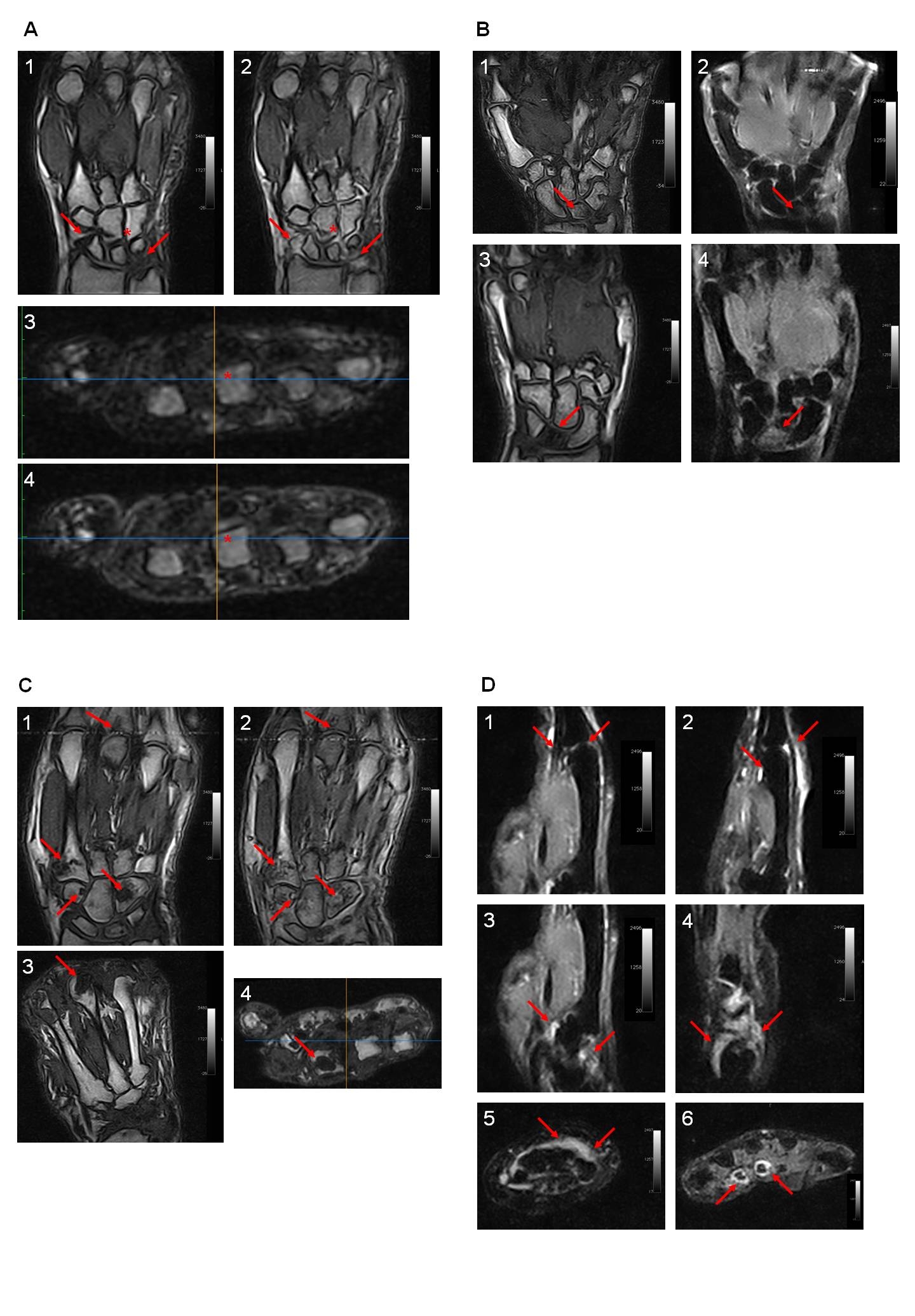

Supplement: Additional file 1 — Figure S1 showing typical examples of arthritis features on low-field MRI. This figure provides typical examples of synovitis, bone marrow edema, erosions, joint effusion, and tenovaginitis on low-field MRI. (A) Synovitis: T1-weighted gradient-echo sequence before (1, 3) and after (2, 4) application of Gadodiamide shows definite (*) synovitis of the intercarpal and radioulnar joints (1, 2) and the third metacarpal joint (3, 4) and severe (arrows) synovitis with synovial thickening and contrast enhancement of the radiocarpal and ulnocarpal joints (1, 2). (B) Bone marrow edema (arrows) affecting < 50% of the bone marrow area (1, 2) and severe edema affecting > 50% of the bone marrow area (3, 4) appear as hypointense areas in T1-weighted images (1, 3) and hyperintense areas in T2-weighted short-tau inversion recovery images (2, 4). (C) Erosion: T1-weighted gradient echo sequence before (1) and after (2) application of Gadodiamide show erosions (arrows) of different sizes. A severe erosion is shown in coronary (3) and transverse (4) sections. (D) Effusion: T2-weighted short-tau inversion recovery image visualizes hyperintense fluid signals (arrows) of normal joint fluid (1), definite effusion (2, 3), and severe effusion (4, 5) as well tenovaginitis of the flexor tendons grade 2 (6). [file ar4128-S1.JPEG]
