# Supplementary material for: Low-field magnetic resonance imaging study on carpal arthritis in systemic sclerosis - low-grade erosive arthritis of carpal bones is an unexpected and frequent disease manifestation
Source: Arthritis Res Ther. 2013 Jan 4;15(1):R2. doi: 10.1186/ar4128 (PMC3672762; doi:10.1186/ar4128)
Supplement: Additional file 2 — Table S1 presenting a summary of the RAMRIS. This table provides an overview of the RAMRIS per patient. N/A, not applicable due to lack of venous access for the injection of contrast agent. [file ar4128-S2.DOC]

**Supplementary Table 1**

| **Patient** | **Hand** | **Synovitis** | **Bone marrow edema** | **Erosion** | **Total RAMRIS** |
| --- | --- | --- | --- | --- | --- |
| 1 | Right | 11 | 0 | 2 | 13 |
| 2 | Left | 1 | 0 | 0 | 1 |
|  | Right | 1 | 4 | 4 | 9 |
| 3 | Left | 2 | 0 | 4 | 6 |
|  | Right | N/A | 3 | 2 | N/A |
| 4 | Left | 7 | 0 | 7 | 14 |
|  | Right | 4 | 1 | 5 | 10 |
| 5 | Right | 8 | 0 | 3 | 11 |
| 6 | Left | 0 | 0 | 4 | 4 |
|  | Right | 0 | 0 | 3 | 3 |
| 7 | Left | N/A | 0 | 2 | N/A |
|  | Right | 2 | 0 | 1 | 3 |
| 8 | Left | 0 | 3 | 3 | 6 |
|  | Right | 1 | 2 | 5 | 8 |
| 9 | Left | 1 | 1 | 2 | 4 |
|  | Right | 9 | 0 | 4 | 13 |
| 10 | Right | 5 | 1 | 3 | 9 |
| 11 | Right | N/A | 1 | 2 | N/A |
| 12 | Right | 2 | 0 | 1 | 3 |
| 13 | Right | 5 | 0 | 3 | 8 |
| 14 | Left | 0 | 0 | 0 | 0 |
| 15 | Right | 0 | 0 | 2 | 2 |
| 16 | Right | N/A | 0 | 2 | N/A |
| 17 | Left | 0 | 0 | 8 | 8 |
|  | Right | N/A | 0 | 4 | N/A |
| 18 | Right | 3 | 2 | 7 | 12 |
| 19 | Left | 6 | 2 | 6 | 14 |
|  | Right | 3 | 6 | 2 | 11 |
| 20 | Left | 0 | 0 | 2 | 2 |
|  | Right | 4 | 0 | 5 | 9 |
| 21 | Right | 0 | 0 | 0 | 0 |
| 22 | Right | 0 | 0 | 1 | 1 |
| 23 | Right | 0 | 0 | 1 | 1 |
| 24 | Right | 0 | 0 | 0 | 0 |
| 25 | Left | N/A | 0 | 1 | N/A |
|  | Right | N/A | 1 | 2 | N/A |
| 26 | Left | 0 | 0 | 4 | 4 |
|  | Right | 4 | 3 | 7 | 14 |
